# A Novel *In Vitro* Protocol for Inducing Direct Somatic Embryogenesis in *Phalaenopsis aphrodite* without Taking Explants

**DOI:** 10.1155/2014/263642

**Published:** 2014-05-15

**Authors:** Jia-Hua Feng, Jen-Tsung Chen

**Affiliations:** Department of Life Sciences, National University of Kaohsiung, Kaohsiung 811, Taiwan

## Abstract

An alternative *in vitro* protocol for embryo induction directly from intact living seedlings of *Phalaenopsis aphrodite* subspecies *formosana* was established in this study. Without the supplementation of plant growth regulators (PGRs), no embryos were obtained from all the seedlings when cultured on the solid medium. In contrast, embryos formed from the seedlings on the 2-layer medium and the 2-step culture system without the use of PGRs. It was found that the age of the seedlings affected embryo induction. The 2-month-old seedlings typically had higher embryogenic responses when compared with the 4-month-old seedlings in the 2-layer medium or 2-step system. For the 2-month-old seedlings, 1 mg/L TDZ resulted in the highest number of embryos at the distal site of the shoot. However, on the leaves' surface, 0.5 mg/L TDZ induced the highest number of embryos. When the 2-month-old seedlings were cultured using the 2-step method at 1 mg/L of TDZ, the highest embryogenic response was obtained, with an average of 44 embryos formed on each seedling. These adventitious embryos were able to convert into plantlets in a PGR-free 1/2 MS medium, and the plantlets had normal morphology and growth.

## 1. Introduction

In plant tissue culture, the selection of suitable types and sources of explants are critical factors for obtaining a successful culture [1, 2]. Conventionally, wounding the surface of explants can initiate callus tissues or direct regeneration through organogenesis or embryogenesis in* in vitro* culture [1, 3]. However, the browning or blackening of cultures is an obstacle in the establishment of explants, chiefly because of the phenol-like or oxidized compounds that are secreted from wounded tissues [4, 5]. Tissue darkening is a major problem in orchid tissue culture, despite treatments such as the addition of adsorbents and antioxidants, selection of explant types, and shortening of subculture periods [1, 6, 7], which inhibit callus formation or regeneration from explants in several types of orchids [6–13]. In the previous works on* Phalaenopsis* orchids, light induced explants to secrete toxic substances and gave an almost complete inhibition on direct embryo induction [12–14].

Plants have the ability to propagate though asexual means, such as vegetative apomixis or via* in planta* somatic embryogenesis, as long as the parent plant is still alive [15, 16]. In this study, we proposed an alternative* in vitro* protocol for embryo regeneration directly from intact living seedlings to avoid wounding tissues and consequently reduce tissue darkening.

To our best knowledge, the species used this present study;* Phalaenopsis aphrodite* subspecies* formosana* (formerly* P. amabilis* variety* formosa* Shimadzu) is qualified to be a model plant in the recent orchid research which has been intensively studied in the past 10 years, including* in vitro* protocols, flowering and photosynthetic physiology, chloroplast genomic analysis, global analysis of transcriptome, and modified ABCDE model of flowering [11, 17–28]. In the global horticultural trade,* Phalaenopsis* (i.e., moth orchids) is one of the most popular plants in the production of pot plants and cut flowers. It is mainly due to their beautiful flowers, ease of cultivation in the artificial conditions, and a long vase life [18, 25, 27]. Since the 1990s, their production has become an important commercial industry in Taiwan and also other countries. The plant material used in this study,* P. aphrodite* subsp.* formosana*, is a native of Taiwan and has excellent flowering characteristics with large moth-shaped flowers that are pure white, in addition to its vigorous vegetative growth [18, 20, 25]. Therefore, it has been used as parent plants extensively in the breeding program of large floral* Phalaenopsis* hybrids and lots of popular commercial cultivars throughout the world [23].

## 2. Materials and Methods

### 2.1. Plant Materials

**Figure 1:**
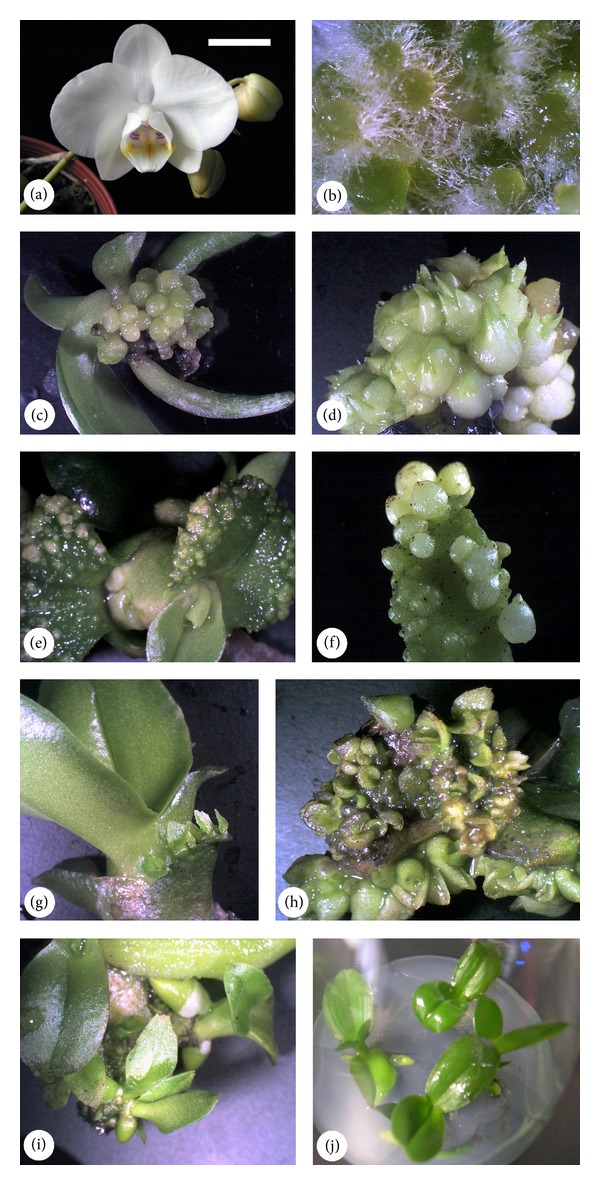
The donor plants of* P. aphrodite* subsp.* formosana*, their seed germination, and direct somatic embryogenesis from intact seedlings. (a) A flowering potted plant for self-pollination to obtain seeds (*bar* 1.5 cm). (b)* In vitro* seed germination after 1 month of sowing on 1/4 MS medium (*bar* 1 mm). (c) A cluster of embryos originated from the posterior region of a 2-month-old seedling (*bar* 3 mm). (d) The embryos turned into protocorm-like bodies (*bar* 2.5 mm). (e) The early event of embryogenesis originated from the leaf surface of a 2-month-old seedling (*bar* 3.5 mm). (f) The foliar embryos formed from an intact 2-month-old seedling (*bar* 2 mm). (g) The foliar embryos developed leaves while they were still connected with the first leaf of the parent seedling (*bar* 5 mm). (h) Numerous embryos initiated from almost the entire seedling (bar = 4 mm). (i) Plantlet conversion while the embryos were still on the parent seedling (*bar* 4.5 mm). (j) Two-month-old rooted plantlets were successfully established (*bar* 1 cm).

Green capsules were collected from* P. aphrodite* subsp.* formosana* (Figure 1(a)) potted plants following 120 d of self-pollination. The capsules were wiped with 75% ethanol and agitated for 15 min in a solution of 0.6% sodium hypochlorite with several drops of Tween 20. After being washed with distilled water 3 times, the seeds were taken from the capsules and sown on a 1/4 strength MS [29] medium supplemented with 1 g/L peptone (Becton, Dickinson and Company, Sparks), 5 g/L coconut powder (PhytoTechnology Laboratories, Shawnee Mission), 20 g/L sucrose, and 8.5 g/L Bacto agar (Becton, Dickinson, and Company, Sparks). Thereafter, the cultures were incubated in 250 mL flasks. The seeds germinated (Figure 1(b)) and the 2-month-old seedlings (approximately 0.5 cm in height) with 2 leaves and the 4-month-old seedlings (approximately 1 cm in height) with 4 leaves were used for testing.

### 2.2. Culture Conditions

The pH of the media was adjusted to 5.2 with 1N KOH or HCl prior to autoclaving for 15 min at 121°C. All the cultures were placed in an incubator with a 16 : 8 h light/darkness photoperiod at 28–36 *μ*mol m^−2^ s^−1^ was used, and the temperature was 25 ± 1°C.

### 2.3. Induction of Embryogenesis in the Three Culture Systems

BA (N^6^-benzyladenine) at 1, 2.5, and 5 mg/L or TDZ [1-phenyl-3-(1,2,3-thiadiazol-5-yl)-urea] at 0.1, 0.5, and 1 mg/L was added to test the effects on direct embryogenesis from the living seedlings in the three culture systems. A plant growth regulator-free (PGR-free) treatment was the control. The additional culture conditions for the 3 culture systems are listed below.

Solid medium: the seedlings were cultured vertically, and approximately 1/5 of the roots were immersed in the solid medium containing 1/2-strength MS basal macro- and micronutrients and full-strength vitamins and supplemented with 1 g/L peptone, 20 g/L sucrose, and 4 g/L Gelrite. The cultures were incubated in 125 mL flasks. The total culture period was 4 months, and the interval of the subculture was 2 months.

Two-layer medium: this medium consisted of an upper layer with a 1 cm height for the liquid medium (which had the same composition as the solid medium but did not contain a gelling agent) and a solid-medium lower layer. The seedlings were cultured vertically with approximately 1/5 of the roots immersed in the solid medium, with the rest of the seedlings completely immersed in the upper liquid medium. The culture period was 4 months, and the interval of the subculture was 2 months.

Two-step culture system: first, the seedlings were cultured in the liquid medium for 2 months. The media for liquid cultures were the same as those used for the solid cultures but without Gelrite. The seedlings were completely immersed in the medium. In addition, the cultures were exposed to an orbital shaker (Scilab Instruments Co., LTD, 110 Taiwan) at 120 rpm for air exchange. Thereafter, the seedlings were transferred to the 2-layer medium without shaking for additional 2 months of culturing. The culture period was 4 months, and the interval of subculture was 2 months.

### 2.4. Culturing of the Plantlets

The embryo-derived plantlets were transferred onto a 1/4 MS medium with 20 g/L sucrose, 1 g/L peptone, 1 g/L active charcoal, and 8.5 g/L Bacto agar for the further development and were given bimonthly subcultures until they matured.

### 2.5. Experimental Design

Twenty seedlings were incubated in 5 flasks for each treatment. Each flask that contained 4 plants had one replicate, and each treatment had 5 replicates. The cultures were incubated according to a completely random design to study the effects of the factors.

### 2.6. Measurements and Data Analysis

The structure that was approximately 3-4 mm in diameter and consisted of the scale leaves was counted as one embryo. The number of embryos per seedling for each treatment was recorded following 4 months of culturing at 2 sites of the seedling, including the distal shoot and leaf surface, with a stereomicroscope (SZH, Olympus, Japan). All treatment means were compared by following Duncan's Multiple Range Test [30]. Significant differences between means were presented at the level of  *P* ≤ 0.05.

## 3. Results and Discussion

### 3.1. Embryo Induction on the Solid Medium

For the PGR-free 1/2MS solid medium, both the 2- and 4-month-old seedlings grew and developed normally without forming adventitious embryos. In contrast, the BA at 1, 2.5, and 5 mg/L and TDZ at 0.1, 0.5, and 1 mg/L induced embryo formation from the distal site of shoots of the 2- (Table 1) and 4-month-old seedlings (Table 2). However, even with the BA and TDZ, no embryos were induced from the leaf surfaces of the seedlings of either age (Tables 3 and 4). As shown in a previous report, the protocorms of* P. aphrodite* subsp.* formosana* have the ability to form secondary protocorms in a PGR-free 1/2 MS medium. The origin of repetitive embryogenesis was primarily the epidermal cell layer of the posterior region of the protocorm [17]. In this study, we found that the epidermal cells on the distal site of the shoots of the 2- and 4-month-old seedlings did not have embryogenic competence when cultured in a PGR-free 1/2 MS medium. Therefore, the seedling age was suggested to be a crucial factor for the induction of embryos.

The TDZ at 3 mg/L induced the highest number of embryos from the protocorms [17]. However, in this present study, a lower dose of TDZ (0.5 or 1 mg/L) was better suited for inducing embryogenesis from seedlings (which are older than protocorms), suggesting that the concentration of the growth regulator for embryo induction depends on age.

Leaf segments have been used as explants to regenerate several types of orchids, including* Dendrobium* [10],* Phalaenopsis* ([11, 13, 14], and* Oncidium* [31]. An adequate concentration of cytokinin typically induces explants to form embryos. However, in this study, BA and TDZ had no positive effects on embryo induction from the leaf surfaces of the intact seedlings when cultured in a solid medium (Tables 3 and 4). The leaf surfaces of seedlings did not direct contact with the medium, and thus it may minimize the effects of PGRs. In addition, the homeostatic regulation of hormones in the intact seedlings may have diluted or neutralized the effects of the exogenous cytokinins.

### 3.2. Embryo Induction in the 2-Layer Medium

To ensure direct contact between the seedlings and medium, a solid/liquid 2-layer medium was used. In the solid culture, the PGR-free treatment did not induce embryogenesis from the distal sites of the shoots (Tables 1 and 2). However, the 2-layer medium system induced embryogenesis from the distal sites of the shoots in the PGR-free treatment (Tables 1 and 2). It was suggested that the waterlogged condition provided by the upper liquid medium may have disrupted the hormone or physiological balance of the seedlings, enabling the cells at the distal sites to express the embryogenic competence. The TDZ of 0.5 mg/L significantly promoted embryogenesis from the distal site of the 2-month-old seedlings, compared to the PGR-free treatment (Table 1). However, in the 4-month-old seedlings, TDZ at 0.1 mg/L and BA at 1 mg/L significantly increased embryo numbers at the distal site of the shoot (Table 2). For the Dendrobium, the 2-layer medium was used during the induction procedure to stimulate early flowering [32]. However, our results showed no early flowering for the cultures in the 2-layer medium.

The leaf surfaces of the 2-month-old seedlings had the ability to form embryos in the PGR-free treatment using the 2-layer culture system (Table 3). An interesting adaptation of the bog adder's-mouth orchid (*Malaxis paludosa*) is its ability to develop small vegetative foliar embryos in its leaf margins that can become new plants [15]. In this study, we found that the waterlogging effect and a direct contact with the medium provided by the 2-layer medium system were crucial for releasing the embryogenic competences of the leaf cells of* Phalaenopsis*. However, no embryos were found on the leaf surfaces of the 4-month-old seedlings in the PGR-free treatment using the 2-layer culture system (Table 4). Therefore, this treatment method did not ensure more differentiated leaf cells for the 4-month-old seedlings across the threshold to form embryos. By adding BA and TDZ, the leaf surfaces of the 4-month-old seedlings were stimulated to form embryos in the 2-layer culture system (Table 4). The highest ratio of embryos/seedling was found at 0.5 mg/L TDZ in both seedling ages (Tables 3 and 4).

### 3.3. Embryo Induction by 2-Step Culture System

For the 2-step culture system, the first step was the liquid medium, which may provide stress of waterlogging or agitation for the seedlings. Thereafter, the seedlings were transferred to the second step (2-layer medium). Seedling age highly affected embryogenesis, and the 2-month-old seedlings typically yielded higher amounts of embryos from the distal site (Tables 1 and 2) and the leaf surfaces (Tables 3 and 4) than the 4-month-old seedlings did. The embryogenic responses with BA and TDZ were substantially less in the 4-month-old seedlings than in the 2-month-old seedlings, possibly because the level of differentiation of the somatic cells was higher in 4-month-old seedlings. Therefore, the 2-month-old seedlings were more suitable for inducing embryogenesis than were the 4-month-old seedlings.

For the 2-month-old seedlings, the TDZ at 1 mg/L had the highest number of embryos per seedling, with 28.8 embryos at the distal site (Table 1). However, on the leaf surface, 0.5 mg/L of TDZ induced the highest embryogenic response, with 22.6 embryos per seedling (Table 3). For the PGR-free treatment of the 2-month-old seedlings, the 2-step culture system increased the number of embryos/seedling by more than 6-fold on the leaf surfaces, compared to the 2-layer medium system (Table 3). This indicated that the conditions of agitation and waterlogging provided by the 2 months of liquid culturing may ensure that the leaf cells released their embryogenic competence.

In plant tissue culture, phenolic compounds, especially oxidized phenolics, typically inhibit* in vitro* growth and proliferation [4, 33]. When the explants were excised, the contents of the wounded cells mixed and the phenolic compounds became oxidized, resulting in toxic secretions into the culture medium and, subsequently, the necrotic browning of the cultures [4, 34]. The level of phenolic compounds, such as flavonols, in cells is determined by light and other factors [4, 35, 36]. For the* Phalaenopsis* leaf cultures, light caused severe browning and necrosis of the explants and completely retarded direct somatic embryogenesis [11, 13, 14]. In this present study, the induction of direct somatic embryogenesis in the intact seedlings was successful when performed in a lighted condition, indicating that somatic embryogenesis without wounding tissues could avoid the browning and necrosis induced by the light regime.

### 3.4. Regeneration Pathway and Plantlet Conversion

The somatic embryos originated primarily from 2 sites: the distal site of shoots and the leaves' adaxial surfaces. For the distal sites of the shoots, the embryos formed nearly simultaneously with the epidermal cells became aggregates and were easily detached from the parent seedlings (Figure 1(c)). Regardless of whether the embryos detached, they had the potential to develop into protocorm-like bodies with scale leaves (Figure 1(d)). For the leaf surfaces, most of the embryos formed from the adaxial site of the explant and developed asynchronously (Figures 1(e) and 1(f)). They followed the same developmental pathway as previously reported [11]. The embryos grew (Figure 1(g)), formed roots, and converted into plantlets (Figure 1(i)) as long as the parent seedling was alive. Under optimal conditions, the embryos formed throughout the leaves' adaxial surfaces and provided high outputs of vegetative propagules (Figure 1(h)). When transferred to the PGR-free 1/2 MS solid medium, all of these plantlets were normal and healthy (Figure 1(j)).

## 4. Conclusion

To our best knowledge, this present study is the first to establish an efficient* in vitro* protocol for inducing direct somatic embryogenesis from intact living seedlings without taking explants. Several treatments, including agitation, waterlogging, BA, and TDZ, were found to be effective in embryo induction from intact seedlings of* P. aphrodite* subsp.* formosana*. The suitable conditions included the following: (1) seed germination in a 1/4 MS medium with 5 g/L coconut powder and 1 g/L peptone to obtain donor seedlings; (2) the 2-month-old seedlings were better than the 4-month-old seedlings for inducing embryos; (3) embryo induction using the 2-step culture system for 2 months followed by culturing on a 2-layer medium for additional 2 months; and (4) 1 mg/L TDZ had the highest embryogenic response with an average of 44 embryos (28.8 embryos on the distal site plus 15.2 embryos on the leaf surfaces) per 2-month-old seedling.

## Figures and Tables

**Table 1:** Number of embryos formed on the distal site of shoots of 2-month-old seedlings, depending on culture systems and concentration of cytokinins.

PGR treatments (mg L^−1^)	Solid medium	Two-layer medium	Two-step culture
PGR-free	0^d∗^	3.6^b^	5.0^b^
BA			
1	5.8^c^	4.0^b^	7.0^b^
2.5	7.4^bc^	7.6^ab^	10.4^b^
5	5.6^c^	5.6^b^	7.8^b^
TDZ			
0.1	8.2^bc^	6.8^ab^	19.0^ab^
0.5	15.4^a^	15.0^a^	16.6^ab^
1.0	9.2^b^	12.4^ab^	28.8^a^

*Data in the same column followed by the same letters are not significantly different by Duncan' multiple range test at *P* < 0.05.

**Table 2:** Number of embryos formed on the distal site of shoot of 4-month-old seedlings, depending on culture systems and concentration of cytokinins.

PGR treatments (mg L^−1^)	Solid medium	Two-layer medium	Two-step culture
PGR-free	0^c∗^	5.6^b^	0^c^
BA			
1	2.4^abc^	11.8^a^	5.2^a^
2.5	2.2^abc^	10.0^ab^	1.6^bc^
5	1.6^bc^	8.8^ab^	1.6^bc^
TDZ			
0.1	3.2^abc^	11.8^a^	2.4^bc^
0.5	6.6^a^	10.2^ab^	3.0^ab^
1.0	6.2^ab^	10.8^ab^	2.2^bc^

*Data in the same column followed by the same letters are not significantly different by Duncan' multiple range test at *P* < 0.05.

**Table 3:** Number of embryos formed on the leaf surface of 2-month-old seedlings, depending on culture systems and concentration of cytokinins.

PGR treatments (mg L^−1^)	Solid medium	Two-layer medium	Two-step culture
PGR-free	0^a∗^	2.6^a^	19.0^a^
BA			
1	0^a^	3.6^a^	20.6^a^
2.5	0^a^	4.6^a^	10.2^a^
5	0^a^	5.0^a^	19.4^a^
TDZ			
0.1	0^a^	6.0^a^	16.2^a^
0.5	0^a^	7.0^a^	22.6^a^
1.0	0^a^	5.6^a^	15.2^a^

*Data in the same column followed by the same letters are not significantly different by Duncan' multiple range test at *P* < 0.05.

**Table 4:** Number of embryos formed on the leaf surface of 4-month-old seedlings, depending on culture systems and concentration of cytokinins.

PGR treatments (mg L^−1^)	Solid medium	Two-layer medium	Two-step culture
PGR-free	0^a∗^	0^c^	5.0^b^
BA			
1	0^a^	2.6^bc^	10.2^ab^
2.5	0^a^	3.6^b^	12.8^ab^
5	0^a^	3.0^bc^	8.0^b^
TDZ			
0.1	0^a^	5.0^ab^	15.2^ab^
0.5	0^a^	7.0^a^	19.2^a^
1.0	0^a^	4.4^ab^	12.6^ab^

*Data in the same column followed by the same letters are not significantly different by Duncan' multiple range test at *P* < 0.05.
